# An interpretable machine learning approach to study the relationship beetwen retrognathia and skull anatomy

**DOI:** 10.1038/s41598-023-45314-w

**Published:** 2023-10-24

**Authors:** Masrour Makaremi, Alireza Vafaei Sadr, Benoit Marcy, Ikram Chraibi Kaadoud, Ali Mohammad-Djafari, Salomé Sadoun, François De Brondeau, Bernard N’kaoua

**Affiliations:** 1 Dentofacial Orthopedics Department (UFR de Sciences Odontologiques), University of Bordeaux, Bordeaux, France; 2 Bordeaux Population Health (Team ACTIVE), INSERM U1219, University of Bordeaux, Talence, France; 3 Department of Public Health Sciences, College of Medicine, The Pennsylvania State University, Hershey, PA 17033 USA; 4 Theoretical Physics Department, University of Geneva, 1211 Geneva, Switzerland; 5 University of Toulouse, Toulouse, France; 6 LUSSI Department, IMT Atlantique, 29238 Brest, France; 7 LSS, Centrale Supelec, Paris, France

**Keywords:** Computational biology and bioinformatics, Evolution, Biomarkers, Computer science

## Abstract

Mandibular retrognathia (C2Rm) is one of the most common oral pathologies. Acquiring a better understanding of the points of impact of C2Rm on the entire skull is of major interest in the diagnosis, treatment, and management of this dysmorphism, but also permits us to contribute to the debate on the changes undergone by the shape of the skull during human evolution. However, conventional methods have some limits in meeting these challenges, insofar as they require defining in advance the structures to be studied, and identifying them using landmarks. In this context, our work aims to answer these questions using AI tools and, in particular, machine learning, with the objective of relaying these treatments automatically. We propose an innovative methodology coupling convolutional neural networks (CNNs) and interpretability algorithms. Applied to a set of radiographs classified into physiological versus pathological categories, our methodology made it possible to: discuss the structures impacted by retrognathia and already identified in literature; identify new structures of potential interest in medical terms; highlight the dynamic evolution of impacted structures according to the level of gravity of C2Rm; provide for insights into the evolution of human anatomy. Results were discussed in terms of the major interest of this approach in the field of orthodontics and, more generally, in the field of automated processing of medical images.

## Introduction

With an incidence of 44% among 12-year-old children, Class II by mandibular retrognathia (C2Rm) is the most common craniofacial dysmorphosis^1,2^ with an unequal distribution according to sex and ethnicity^3^ as well as a hereditary component. This pathophysiology is characterized by a too short mandible that leads to a loss of occlusion with the maxilla. Conversely, prognathism corresponds to the situation where either the maxilla or the mandible is positioned forward compared to normal positioning.

Retrognathia, and malocclusions in general, are associated with changes in craniofacial morphology. For example, a link between fusions of cervical vertebrae, deviations in the cranial base and mandibular retrognathia has been documented radiographically^4^. Indeed, the association between the angle of the cranial base and the morphology of the cervical spine plays a central role in establishing the diagnosis of malocclusion. These morphological changes are also important to consider when searching for etiology, especially in cases of severe skeletal malocclusions^5^.

Overall, the morphology of the face depends on many factors such as sex, ethnicity, climate, nutrition, genetic constitution, etc^6^. Regarding ethnicity, for example, cephalometric analysis of Bangladeshi and Japanese adults^7^ showed that the mandibular antero-posterior position among the Bangladeshi was more protrusive compared with that of Japanese subjects. Proffit et al.^8^ found that the SNA angle (the angle between the sella, nasion and anterior nasal spine) was significantly greater in African Americans than in Caucasians, resulting in a more prognathic facial profile in African Americans compared to Caucasians. The results of these studies are valuable for orthodontics, insofar as the analysis of craniofacial morphology provides information on the typology of the patient, on the shift between the bony bases, as well as on the diagram of mandibular growth. These elements (in particular, the extent of malocclusion and the changes in craniofacial morphology) constitute the basis of the diagnosis and make it possible to develop a treatment strategy, or a treatment plan, adapted to each type of malocclusion, according to its importance and its orientation.

Moreover, the understanding of C2Rm represents an anthropological challenge insofar as Retrognathia is at the heart of the evolution of hominins^9–12^.

Since the differentiation of the genus Homo, a concomitant kinematics of transition from more prognathic forms to more retrognathic faces has been observed^13^ but its phylogenetic and ontogenetic mechanisms are still unknown. This decrease in mandibular length was accompanied by an increase in brain volume. Even if the relationship between the length of the mandible and the craniofacial architecture remains to be clarified^14^, the transition from prognathism to an increasingly accentuated retrognathia has been accompanied by a series of craniofacial modifications central to human evolution^11^. Mandibular growth is sensitive to food consistency and changes in occlusal forces (mastication/breathing/swallowing). However, the industrial revolution led to a new change in eating habits with the emergence of processed products, mostly soft, and a more sedentary lifestyle. These changes caused an imbalance in the forces experienced by the skull during its development, which ultimately led to an increase of the number of cases of C2Rm and of their severity^15^. A better understanding of the craniofacial changes induced by the increase in mandibular retrognathia therefore appears to be a good model for studying the craniofacial evolution of hominins.

**Figure 1:**
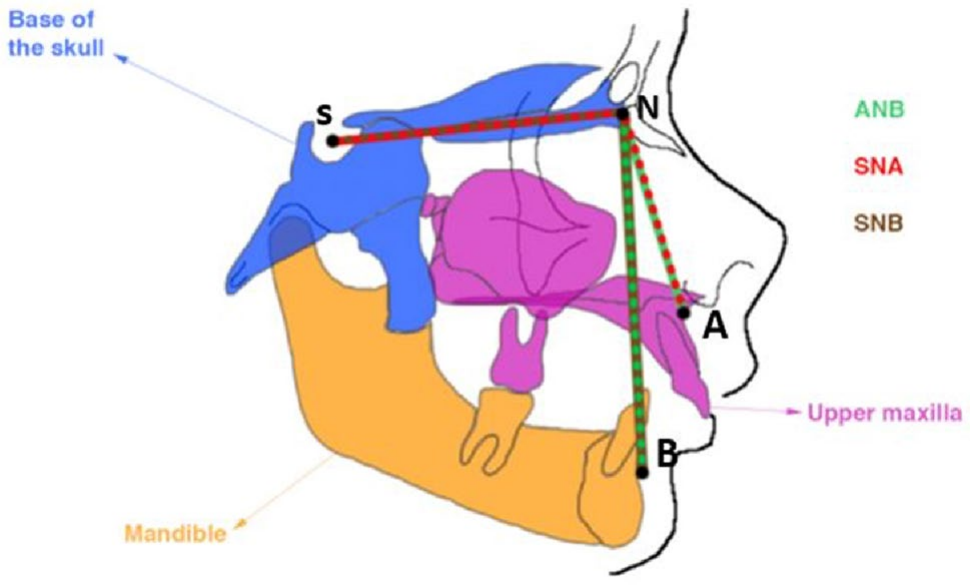
Retrognathia and the three standard cephalometric angles widely used in evaluating anteroposterior apical base relationship: ANB in green, SNA in red, SNB in brown (ANB = SNA–SNB). The unit of measurement of angles is the degree.

In orthodontic practice, the sagittal relationship between the maxilla and the mandible is an important diagnostic criterion for C2Rm. A classical parametric model with three standard cephalometric angles is widely used to characterize this relationship^16^: the Maxillary-Nasion-Mandibular (ANB) angle, the Sella-Nasion-Maxillary (SNA) and Sella-Nasion-Mandibular (SNB) angles. As illustrated in Fig. 1, the ANB angle expresses the relative position of the maxilla to the mandible, whereas the SNA and SNB angles indicate respectively the anteroposterior projection of the maxilla and the mandible.

Class II by mandibular retrognathia is characterized by an increased ANB angle (ANB [> 4]) and a decreased SNB angle (SNB [< 76]). The increased ANB angle implies that the mandible occupies a retrusive position in relation to the maxilla, taking as a reference the base of the skull. The reduction in the SNB angle induces that the mandible is in a retrusive position relatively to the base of the skull.

While being widely used in orthodontic practice, these cephalometric angles present some major limits. First, they focus on the jawbone, and therefore fail to highlight the rest of the structures potentially impacted by C2Rm physiopathology. Regarding this limit, decades of research based on cephalometric parameters investigated craniofacial alterations correlated to C2Rm. These studies aim to identify the impact of C2Rm on the shape of the skull, but also to predict the pattern of mandibular growth. Indeed, growth prediction plays a significant role in accurate diagnosis and treatment planning of orthodontics patients. For example, it has been considered that the unique pattern of pneumatization of the frontal sinus could be used as a predictor of growth^17^. Other statistical correlations have been highlighted, such as cervical vertebrae^18^ or the cranial base^19^, but the results thus obtained vary from one study to another (depending on the sample size, the employed method, etc.). This is partly because the methods typically used rely on biologically relevant landmarks (based on predefined biological knowledge) that are manually assigned to images. This assignment is error-prone and makes it difficult to discover new anatomical features related to retrognathia that may prove relevant in understanding and managing this malocclusion^20^.

These limits have been partly circumvented by the development of geometric morphometrics^21^. Landmark-based geometric morphometric methods involve summarizing shape in terms of a landmark configuration. The direct analysis of these points would not be relevant due to the possible variations in position, orientation or scale of the specimens. Therefore, variations unrelated to shape are mathematically eliminated by superposition methods. The Procrustes approach is one of the superposition methods classically used to quantify the similarity or dissimilarity between two landmark configurations (physiological class I and pathological class II, for exemple) by calculating the Procrustes distance between them^22^.

But, even if the geometric morphometric techniques have found numerous applications in the biological sciences, the limits of this approach have been widely underlined^23^. In particular, errors may be related to the superimposition procedure. For example, Rohlf^24^ shows that in certain circumstances, the variation observed after a Procrustes superimposition is very different from the model on which it was based. And Walker^25^ offered a detailed description of some problems in estimating the pattern of variances and covariances of landmarks after superimposition. Applied to retrognathia, this means that sometimes the mandible position cannot be controlled during the image acquisition process. As a result, its morphological or metric characteristics cannot be compared between individuals since any observed differences would reflect more this methodological bias than a true interindividual variability.

Another important limitation of geometric morphometry applied to the analysis of medical images lies in the difficulty of understanding the dynamic of interactions between structures at different stages of evolution of a physiological or pathological process. For example, Gkantidis and Halazonetis^26^ evaluated the morphological covariation between the face and the basicranium (midline and lateral line) at two specific developmental stages (children and adults). Lateral cephalometric radiographs were digitized and a total of 28 landmarks were placed in three areas (the medial cranial base, the lateral cranial base and the face). Geometric morphometric methods were applied and partial least squares analysis was used to assess the correlation between the three shape blocks. The results highlight the interactions between the structures of the middle cranial base and facial patterns in the group of children, as well as between the lateral elements of the cranial base and facial patterns at a later lifestage, such as during adulthood. Freudenthaler et al.^27^ assessed the relationship between craniofacial shape and malocclusion using geometric morphometry. Cephalometric radiograph tracings of 88 untreated Caucasians (age range: 7–39 years) were assigned to four groups according to the nature of their occlusion.

The results show that the craniofacial shape was clearly associated with dental malocclusion with considerable variations between the four groups. But if these studies have allowed some advances in the field of orthodontics, Lieberman et al.^28^ conclude that, overall, these methods require independent structure analysis and fail to isolate and define the actual interacting morphogenetic units, identify and quantify their direct and indirect interactions, and understand the processes through which they interact.

In this context, it appears relevant to develop new morphometric tools which do not involve defining areas of interest a priori, nor defining shapes using a set of benchmarks, and which allow to follow the dynamic evolution of structures impacted by the severity of mandibular retrognathia.

In recent years, the progress of research in the use of Artificial Intelligence (AI) in orthodontics has been considerable and many applications have been developed. For exemple, Thurzo et al.^29^, in a documented review, mention the use of machine learning algorithms to automate the analysis of dental images, the development of AI tools to detect oral diseases or to optimize diagnosis and treatment planning. In the field of medical imaging, Deep Neural Networks (DNNs) and particularly Convolutional Neural Networks (CNNs) as well as additional interpretable methods of saliency maps such as class activation mapping (CAM) and its variant Score-CAM^30^ have become very popular tools. They are used in particular to locate biomarkers, i.e., observable or measurable signals that reflect the presence, severity or evolution of a disease and that can be considered as discriminating regions in abnormal images. This approach has been applied to various medical fields^31^, such as retinal fundus images^32^, cancer detection^33^, pneumonia detection or Alzheimer’s diagnosis^34^. A recurrent and common result of these studies is that CNNs outperform practitioners in pathology classification^35^.

CAM and Score-CAM, as interpretable methods, can be applied to CNNs to provide visual identification (using a heatmap) of regions of interest (ROI), i.e. areas in medical images provided as input, on which the CNN actually relied to carry out its classification. The regions of the medical images provided as input are thus classified according to color ranging from hot zones (red color, i.e. extremely relevant for classification) to cold zones (blue color) passing through intermediate zones (orange color). Applied to the retrognathia, this means that the CNN performs a classification of the input images according to criteria provided by the practitioner (in this instance, the measurement of the angles ANB [0–5], SNB [< 77], and SNA [79–85]) and that the interpretability methods identify the ROIs actually used by the classifier to distinguish between the input images (in class I physiologic and class II pathological).

It is therefore important to verify that the ROIs identified by the CNN correspond to biomarkers relevant from a medical point of view and already identified in literature. But this approach can make it possible to identify new regions of interest, which could allow for advances in understanding, treating or managing the pathology considered. In this perspective, interpretable AI algorithms become tools for the discovery and extraction of new knowledge, offering an innovative scientific approach to health issues^36,37^.

Despite the limitations that have been noted by members of the scientific community^38^, these methods may serve as tools in the search for information contained in sets of images. The foundations of this approach have been clarified by Wöber et al.^20^ in a study aimed to characterize the phenotype of a fish population based on the animal’s habitat. The authors start from the classic observation that assigning landmarks is tedious and error-prone and that predefined landmarks can miss information that is not obvious to the human eye. They document the new data analysis methods proposed by the machine learning (ML) community to identify subtle features in images, with an excellent predictive accuracy. The authors also insist on the contribution of interpretability methods by means of which it is possible to overcome the problem of the black box of ML methods, by making internal representations (ROI) accessible via saliency maps. The authors then conclude on how deep learning improves traditional morphometric analysis, particularly in terms of predictive performance.

Our work is inscribed in the same perspective, and aims to apply this approach to health, in particular to mandibular retrognathia, which is a central field in orthodontic research.

The main objective of our work was to propose a methodological pipeline coupling CNN and interpretability approaches to extract knowledge concerning the shape of the skull and, in particular, to evaluate the impact of a pathological process (mandibular retrognathia) on the entire skull (this is the first study ever carried out in this area). This objective required the generation of a unique saliency map, named “global activation map”. Taking into account all pathological individuals, this map aimed to crush inter-individual variability and to highlight the biomarkers common to the totality of the study population.

A secondary and complementary objective was to use this methodology to monitor the dynamic evaluation of impacted skull structures according to the severity of retrognathia; towards this end, saliency maps were generated and compared according to different C2Rm severity levels.

## Methods: Morphological information extraction with an Interpretable CNN (MIE-ICNN) Methodology

**Figure 2:**
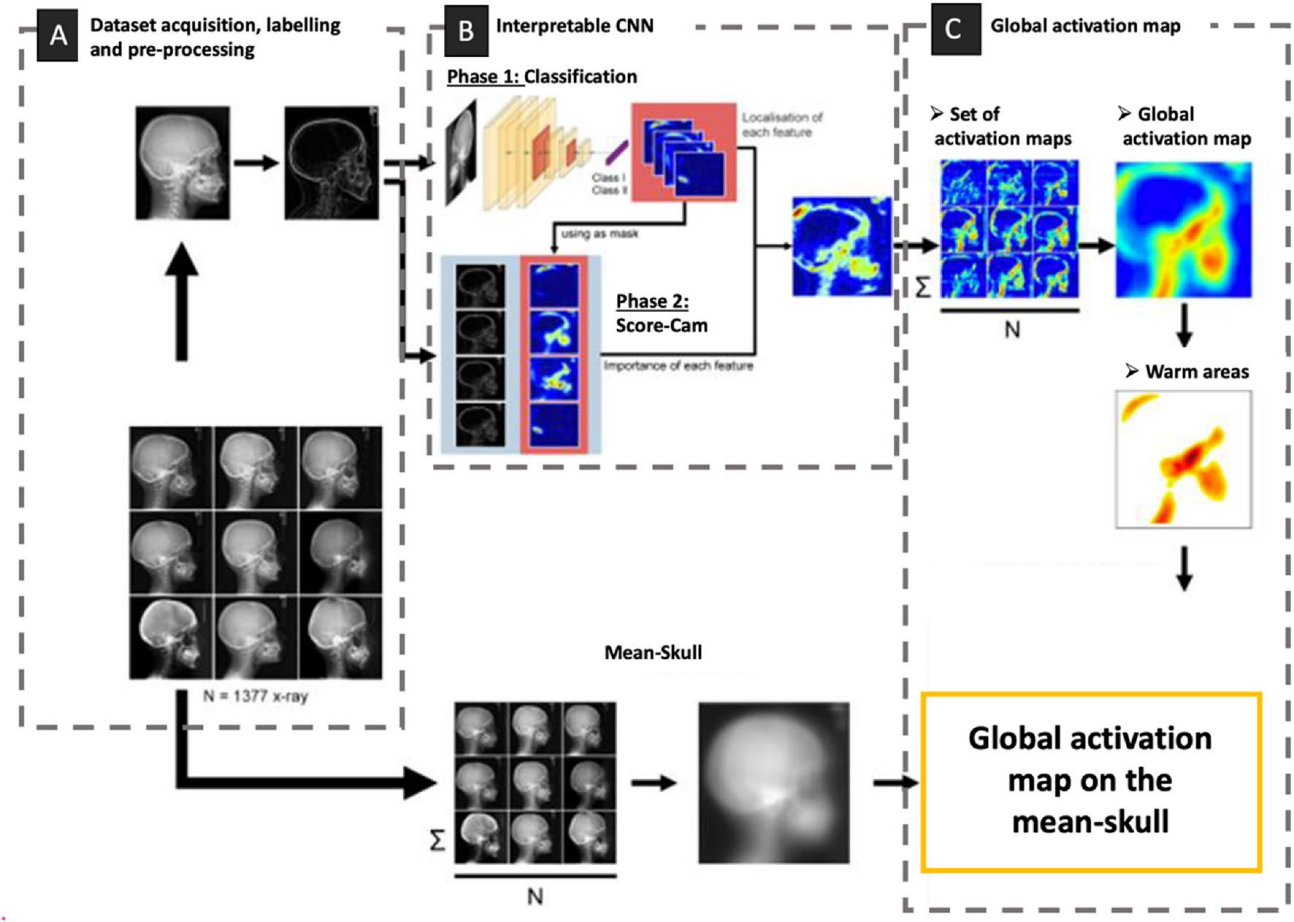
Synthetic presentation of the main steps of the morphological information extraction methodology with an Interpretable CNN (MIE-ICNN) that we used. (**A**) Dataset acquisition, labeling and pre-processing; (**B**) Interpretable Deep CNN training-assessment-testing process; (**C**) Generation of global activation map.

This section presents the main steps of our methodology for extracting morphological information using interpretable convolutional neural networks (MIE-ICNN). The characteristics of the dataset are first presented, encompassing the acquisition, selection and labeling of data as well as the pre-processing step (Fig. 2A). Next, the architectural configuration and parameters of our interpretable CNN (Fig. 2B) and then the method for generating global activation maps are described (Fig. 2C).

### Dataset acquisition and labeling

Our study focused on a compilation of cephalometric images, including 2694 French orthodontic patients. These were patients of both sexes and a wide age range (no selection based on age or sex was made in this study), complaining of dentofacial dysmorphosis and/or malocclusion (1/3 males and 2/3 females with a mean age of 12.63 ± 5.06 years).

These 2694 X-rays were selected from an initial pool of 23,479 X-rays from four separate clinics. To guarantee the fidelity of the data, this initial pool included only radiographs adhering to the standardized positioning of the cephalostat, and coming from the same radiography device (CARESTREAM CS 9000-C).

**Figure 3:**
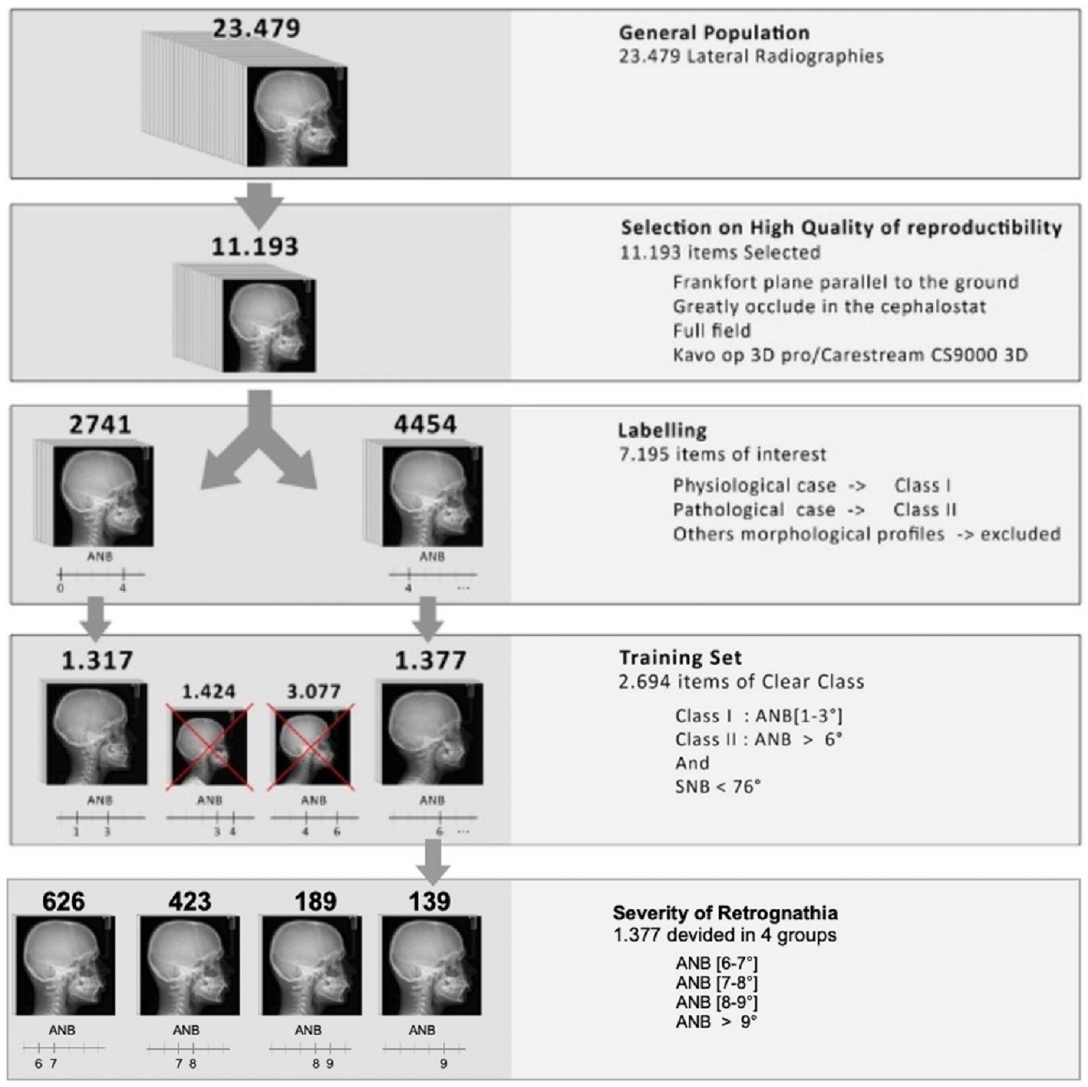
Radiography images selection and labeling process for dataset elaboration.

A meticulous image selection and labeling process, shown in Fig. 3, was then performed. After review by dentofacial orthopedist practitioners, a subset of 11,193 images was chosen based on high quality attributes, adherence to anatomical standards (including Frankfurt plane orientation^39^) and a complete skull X-ray.

Then, only the images showing a Class I and a Class II with a retrognatic mandible were retained (7100 X-ray) and classified into two categories, “physiological” (2741) and “pathological” (4454), according to angle measurements (ANB [> 4] and SNB [< 76]). Images with other types of malloclusion were thus excluded. To reinforce the discrimination between the categories, borderline cases between physiological-pathological (ANB [(− 1) − 1]) were excluded. The final training set included 1317 Class I physiological images and 1377 Class II images.

In a second step, and in order to evaluate the modifications of the shape of the skull according to the severity of the retrognathia, the class 2 images were subsampled into 4 categories according to the amplitude of the angle ANB (from [6° to 7° [and gradually increasing to [9° <] in steps of 1). We therefore referred to Steiner’s classification which defines the extent of retrognathia according to the value of the angle ANB^40^.

The distribution included 626 individuals exhibiting an ANB angle between [6° and 7°[, 423 exhibiting an ANB angle between [7°-8°[, 189 exhibiting an ANB angle between [8°–9°[, and 139 whose ANB angle was comprised between [9° <].

### Pre-processing step

The image preprocessing step plays a crucial role in improving the efficiency of CNN and optimizing the memory consumption of the graphics processing unit (GPU). For this, we performed the following pre-processing operations:

(i) Resizing:

Considering the importance of resizing pre-processing^41^ and variability in our dataset, all X-ray images were standardized to 256 × 256 pixel dimensions. This resizing reduces potential distortions resulting from variability in craniofacial growth patterns (due to age, gender, ethnicity, etc.), thereby focusing attention on pathology-induced disparities.

(ii) Normalization:

To account for sigmoid or softmax functions related to the backpropagation algorithm, we normalized the pixel intensity values to a range between 0 and 1 before entering them into the neural network^42^.

(iii) Sobel filtering:

The use of a Sobel process^43^ enhanced edge detection capabilities by emphasizing edge-related intensity changes.

### Interpretable deep CNN architecture

In this section, we first describe the architecture and parameters of Deep CNN, then the interpretability process that we have implemented.

#### Classification

To extract bone shape features from the lateral radiographies, first, a binary classification model was built and used to classify data in class I and class II as illustrated in Fig. 2B (Phase 1).

To train the model and extract high-level features^44,45^, we utilized a deep-learning model. The model architecture includes seven CNN layers with 867,178 parameters that exhibited the highest accuracy regarding identification of the class, at a percentage of 97%. Each of these layers was paired with a batch normalization and a ReLU activation function^46^.

A SoftMax activation function was used on the last fully connected layer to make the predicted class. The model was fine-tuned using an ADAM optimizer^47^ with a decreasing learning rate starting at 10^–3^ and decreasing by 0.95 per 100 epochs. A total of 1000 epochs was reached with a batch size of 100 in a P100 Nvidia GPU, using 70 GB of memory.

In Deep Learning classification methods, when the number of parameters is large, some form of regularization is needed to ensure small generalization errors and avoid overfitting^48^.

In this context, statistical learning theory offers several different tools capable of controlling and avoiding overfitting. In our study, we increased and dropped data so as to control generalization errors, following the recommendations of Zhang et al.^48^:

(i) Regarding data augmentation, during training, the preprocessed X-rays are augmented by the following operations: random horizontal and vertical translations; rotation with steps of 10 degrees, width and height shift, and zoom within the range of 3%. The rotation of the images was performed based on the center of the image, which is located at pixel coordinates (128, 128). This served as the reference point for the rotation process. The time taken for each rotation, was approximately 0.01 s per image.
(ii) We used a dropout layer with a drop rate of 0.5.

To determine the model performance, we divided the dataset into training and testing subsets with an 80–20 ratio for developing and evaluating our CNN model. We used a 5-step cross-validation approach, which is common practice for datasets of this size. This technique involved splitting the dataset into five subsets, using each subset once as a test set, while the remaining four subsets were used for training in each iteration. In other words, the distribution of classes in each iteration consisted of 1317 samples (1054 for training and 263 for testing) for class I and 1377 samples (1102 for training and 275 for testing) for class II. Using cross-validation resulted in an accuracy rate of 97%.

#### Interpretability through saliency maps

Once the deep CNN trained, we used it for a classification task in order to predict the presence or the absence of a C2Rm.

As illustrated in Fig. 2B (Phase 2), for each image classified as positive to C2Rm, a saliency map is generated using the Score-CAM technique. This step involves the implementation of the Score-Weighted Class Activation Mapping (Score-CAM) technique to generate informative saliency maps. These salience maps highlight the craniofacial structures that significantly influence the CNN classification process.

The Score-CAM technique works as a multi-step process, implementing mathematical procedures to highlight the underlying elements that guide CNN predictions:

(i) Prediction Score Extraction:

The final prediction score (Sc) generated by the CNN represents the network's level of confidence in categorizing a given image, effectively quantifying how certain the network is about its decision.

(ii) Backpropagation of Scores:

The identification of the relevant zones allowing the CNN to carry out its prediction goes through a process of backpropagation. This process requires: (1) quantifying the influence of the activation of each channel on the global prediction score; (2) to apply a function that accentuates regional activations that contribute significantly to the prediction”.

Mathematically, the gradients of the predicted class score (Sc) regarding the activations (Ai) of the last convolutional layer (i) are calculated:

$${G}_{i}=\frac{\partial {S}_{c}}{\partial {A}_{i}}$$

These gradients quantify the influence of each neuron's activation on the overall prediction score.

(iii) Weighted Activation Aggregation:

The weighted activations (Mi) for the last convolutional layer were obtained by multiplying the gradients (Gi) by the ReLU activation of the corresponding neuronal output (Ai). This procedure aims to accentuate the activations that contribute significantly to the prediction:

$${M}_{i}={G}_{i}\times \mathrm{ReLU}({A}_{i})$$

(iv) Saliency Map Generation:

The saliency map (L) was created by calculating the weighted average of the activations (Mi) from all channels using a global average pooling. This average activation value (α) highlights the signifcance of each channel of CNN in influencing the network's decision.

$$\mathrm{\alpha }=\mathrm{ GlobalAvgPool}({M}_{i})$$

The final salience map (S) was obtained by linearly combining the weighted activations (Mi) using the calculated importance (α) as the weighting element. This map captures areas of the image that contribute substantially to CNN’s classification decision:

$$S=\sum (\alpha \times {M}_{i})$$

This Score-CAM methodology was therefore applied to the 377 images labeled as C2Rm, generating a corresponding set of salience maps. The next step consisted of averaging these individual saliency maps.

### Global activation map generation

The last step involved calculating an overarching activation map, referred to as the global activation map (depicted in Fig. 2C). To calculate the global activation map: let N be the number of images, each with its corresponding saliency map If for i = 1,2, …, N; these saliency maps are aligned based on the cranial positions; the global activation map G is obtained by averaging the individual saliency maps:

$$G=\frac{1}{N}\sum_{i=1}^{N}{S}_{i}$$

The map thus obtained constitutes a synthesis of individual salience maps, harmonizing the cranial positions for a coherent analysis. To promote interpretability, this map is projected onto an averaged skull, corresponding to a synthesized representation made from all the input radiographs of our dataset.

### Activation map according to the severity of class II

Four salience maps were then created according to the severity of the pathology, using the same method described above. Each map grouped individuals with ANB angles of [6°–7°], [7°–8°], [8°–9°] and [9° <], in order to study the evolution of the maps of salience as a class II gravity function according to Steiner's classification.

### Quantification of regions of interest

To quantify the areas of interest, we defined 2 metrics that we called “class score” and “hot surface”. In order to calculate these metrics, we used the class scores (non-dimensional and normalized) obtained for each of the pixels via the score-cam method. The pixels below a threshold (0.3) have been eliminated in order to keep only the discriminating pixels. Then the various underlying bone structures were delineated and within these areas were calculated:

- *The class score*: the average of the class scores of all the pixels in the area;
- *The hot surface*: the percentage of the area whose pixels exceed the defined threshold.

These two metrics made it possible to quantify the extent and intensity of the dysmorphosis in the areas of interest highlighted by the global activation maps.

### Ethical approval

The study was approved by the Research Ethics Committee of the University Hospital of Bordeaux (reference number CER-BDX-2023-25). As the study was a retrospective review and analysis of fully anonymized lateral radiographies, the Research Ethics Committee of the University Hospital of Bordeaux (reference number CER-BDX-2023-25) waived the requirement for informed consent. This study was conducted in accordance with the relevant guidelines and regulations.

## Results

### Qualitative analysis: descriptive results analysis of saliency maps

This section presents the results obtained, first, on the whole dataset and, second, on datasets related to the different stages of C2Rm evolution, i.e., for each severity level considered in our study.

#### Global activation map

**Figure 4:**
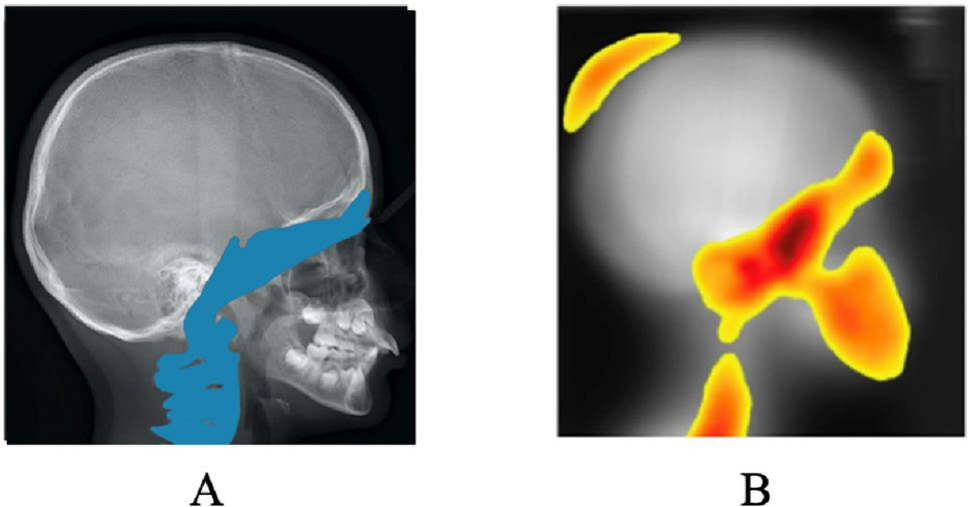
Craniofacial areas: (**A**) Anatomical structure identified in literature as being impacted by mandibular retrognathia: the frontal sinus, the base of the skull, the sphenoid and the cervical vertebrae; (**B**) Anatomical structure highlighted by our global activation map according to MIE-ICNN: the structures already identified in litterature, as well as the parietal bone.

Figure 4A shows the anatomical structures (i.e. biomarkers) identified in literature as being impacted by C2Rm pathology (yellow part of the image). Figure 4B shows the global activation map obtained by the MIE-ICNN method from all class II images used in our study. This global activation map shows, through a heat map, the areas of the images used by the network to issue its prediction. The heat map indicates, using a color code, the importance of the pixels of the image that take part in the prediction of the class. Yellow corresponds to pixels of low importance, orange to pixels of medium importance, and red to pixels of high importance. This system makes it possible to effectively and visually highlight the areas that have been considered important by the classifier in carrying out his classification.

Our results show the presence of different hot zones. Moreover, the analysis of Fig. 4 indicates that all of the highlighted areas appear to be superimposed on bony structures. The area in the upper left is superimposed on the parietal bone. The area in the lower right is centered on the jaws. The third zone is divided into two segments. The first segment covers the cervical vertebrae. It begins at C6 and continues up to the atlas. Activation follows a plane of gravity along this vertebral axis. The inflection point appears to be centered on the sphenoid bone, located in the medial part of the skull base. Finally, the second segment starts at the sphenoid and follows the base of the skull to the frontal sinus.

Our results show that there is a morphotype specific to the population extracted by the deep CNN, which is superimposed on the structures identified in literature (cervical vertebrae, sphenoid, base of the skull to the frontal sinus).

The main novelties here are: (1) the frontal sinus, whose role in C2Rm pathology is debated in literature, and which appears to be discriminating according to our global activation map, (2) the parietal bone, which appears to be discriminating whereas until now it was not cited in literature as an area involved in C2Rm.

#### Saliency map according to the degree of severity of retrognathia

**Figure 5:**
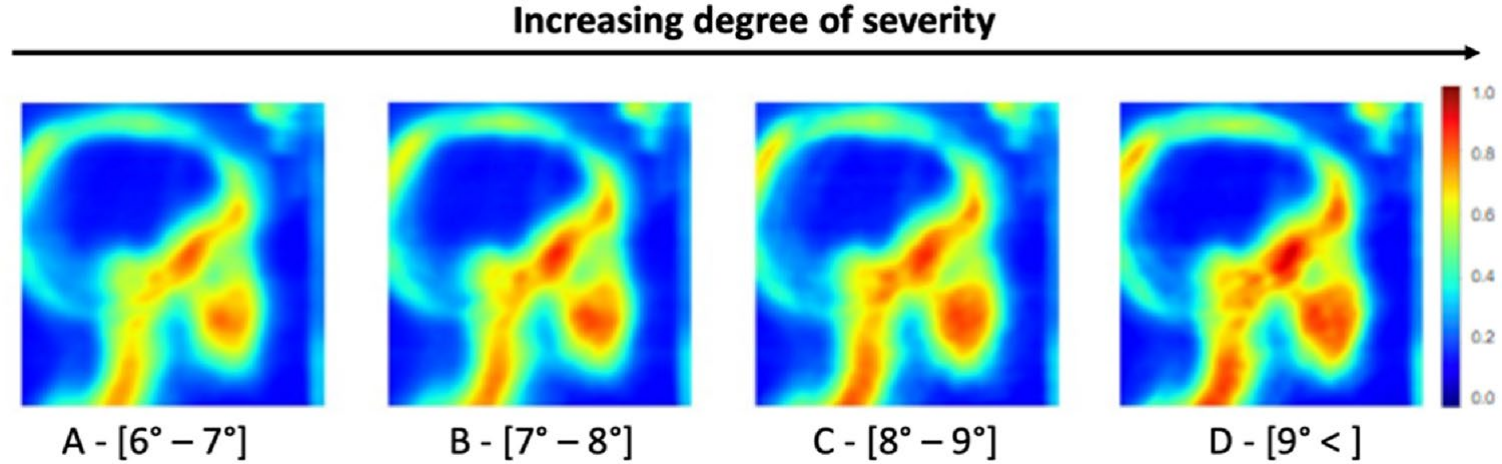
Evolution of the activation map according to the severity of class II. The color bar indicates the class score of the area.

In a second step, we identified the craniofacial structures involved according to the degree of evolution of the pathology using our MIE-ICNN methodology. Figure 5 shows the results of the saliency maps for ANB angles subsampled from [6°–7°] to [9° <] gradually increasing by steps of 1.

**Figure 6:**
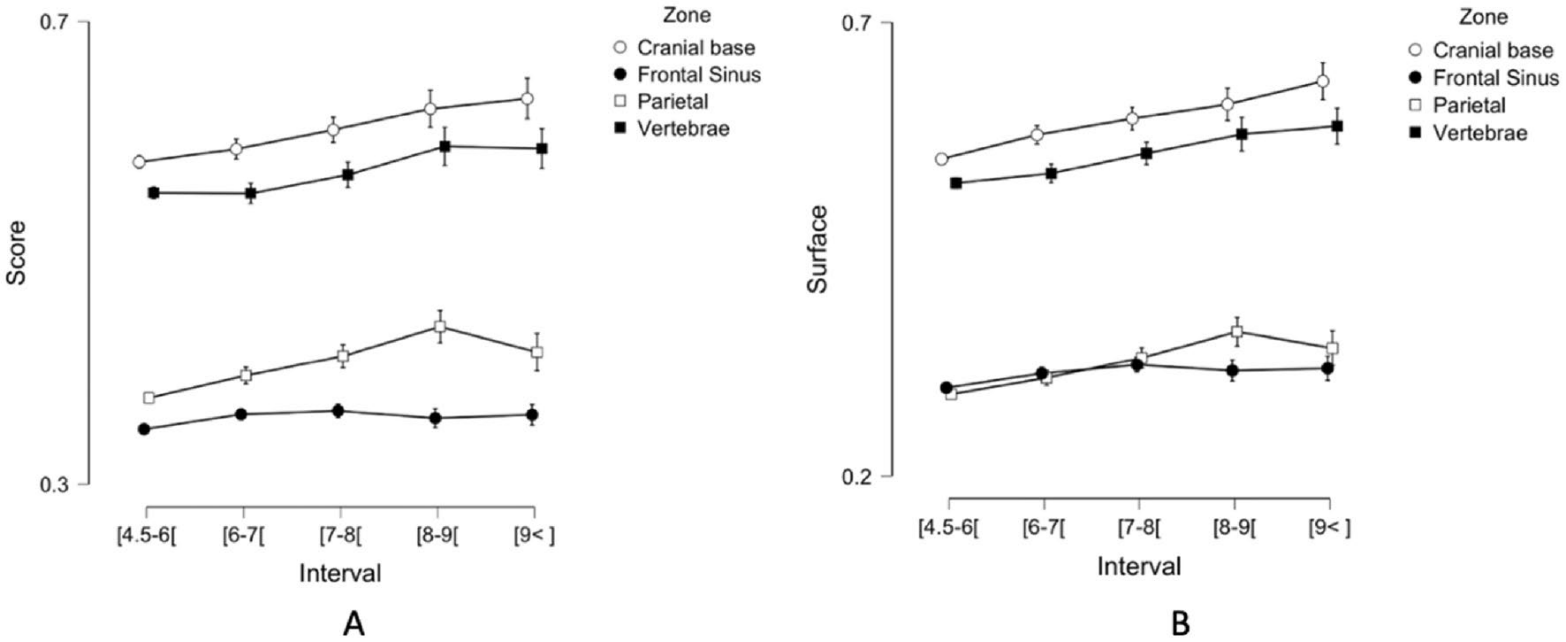
Evolution of metrics according to the ANB angle (Interval) for each of the four skeletal structures (cranial base, frontal sinus, parietal bone, cervical vertebrae): (**A**) Evolution of the class score, (**B**) Evolution of the hot surface.

The analysis of the evolution of the heat map as a function of the severity of C2Rm offers the following results:

The first map (Fig. 6A), shows a global activation the maximum intensity of which is distributed over the bone structures in warm colors (i.e. orange). More precisely, the lower part of the cervical segment, the upper segment up to the sinus, and the mandibular halo appear in orange-red intensity. The rest of the vertebrae and the cranial base, as well as the parietal bone, are shown in yellow. The second map (Fig. 5B) shows an amplification of the intensity of the areas, which get more intense towards the red area. The cervical and basilar segments are now completely red. The diameter of the mandibular halo remains the same, but the red area is intensified. The third map (Fig. 5C) shows the thickening of the cervical-basilar segments and the activation of the inflection point (sphenoid). The mandibular halo continues to extend into the maxilla. The parietal bone changes from yellow to orange. The fourth map presented (Fig. 5D) is characterized by the activation of the parietal bone (orange to red) and the thickening of all other red areas.

Globally, when the amplitude of the angle ANB increases, the results highlight a kinetics of increase in the intensity of the pixels and an expansion of the areas highlighted by the global activation maps.

### Quantitative analysis: statistical results analysis

To assess the impact of increasing ANB angle (retrognathia severity) on saliency maps, we used two metrics: “class score” and “hot surface” (see point 2–6 of the methodology section). Figure 6 presents the results obtained for the four anatomical structures identified above (cranial base, frontal sinus, parietal bone, cervical vertebrae). We performed two 2-way analyses of variance (ANOVA) with ANB angle as between-subjects factor and anatomical structures as within-subjects factor. These two 2-way ANOVAs were conducted with the class score and the hot pixel area as the respective dependent variables. Post hoc comparison has been carried out by using Bonferroni test.

The analyses performed showed a significant effect of the angle factor on both class score F (3, 13,992) = *p* < 0.001 and hot surface F (3, 13,992) = * p* < 0.001 with:

- An increase in class score along the angle: x̄([4.5°–6°[) = 0. 464, x̄([6°–7°[) = 0.474, x̄([7°–8°[) = 0.487, x̄([8°–9°[) = 0.503, x̄([> 9°]) = 0.500);
- An increase in hot surface: x̄([4.5°–6°[) = 0.416, x̄([6°–7°[) = 0.433, x̄([7°–8°[) = 0.451, x̄([8°-9°[) = 0.466, x̄([> 9°]) = 0.471.

The analyses also showed a significant effect of the anatomical structures factor on both the class score F(3, 13,992) = *p* < 0.001 and hot surface F(3, 13,992) = * p* < 0.001 with:

- A higher class score for the cranial base and the cervical vertebrae (x̄(Cranial Base) = 0.588, x̄(Vertebrae) = 0.557) than for the parietal bone and the frontal sinus (x̄(Frontal Sinus) = 0.353, x̄ (Parietal) = 0.387);
- A larger hot surface for the cranial base and the cervical vertebrae (x̄(Cranial base) = 0.566, x̄(Vertebrae) = 0.534) than for the frontal sinus and the parietal bone (x̄(Frontal sinus) = 0.305, x̄(Parietal) = 0.304).

No interaction effect was observed for class score F(12, 13,992) = * p* = 0.379, nor for hot surface F(12, 13,992) = * p* = 0.530.

The lack of significance of the interaction is caused by the fact that the evolution as a function of the angle ANB is the same for the 4 structures.

## Discussion

Recently, AI and, in particular, Machine Learning have come up with new tools that can help detect relevant features in images, bypassing the limitations of geometric morphometry. Moreover, interpretability methods based on the study of internal representations via saliency maps make AI accessible by explaining the elements used by the classifier to issue his prediction. In this study, we applied this innovative approach to mandibular retrognathia, a major health issue in the field of orthodontics.

In this context, we implemented an innovative interpretable AI methodology on orthodontic images by also proposing new elements such as extraction of average saliency maps (on all the individuals concerned) as well as quantification metrics of the ROIs identified by the CNN.

Our main objective was a double one. First, we intended to demonstrate that this methodology was appropriate for identifying image elements allowing a better understanding of the impact of retrognathia on the entire skull; second, we endeavoured to show that it made it possible to monitor the dynamic evolution of impacted cranial structures depending on the severity of retrognathia.

The results obtained are not only in line with these objectives—they also represent arguments in the debate on modifications to cranio-facial morphology during human evolution (linked to the progressive increase in retrognathia). Our discussion will focus on these different areas of contribution of our research.

### Interpretable AI methodology and orthodontic images

In the field of interpretable DNNs, the main contribution of our work is to propose a method of Morphological Information Extraction with Interpretable CNN (which we have called MIE-ICNN) based on a principle of global activation map. CNNs have already demonstrated their ability to extract very subtle features but often using complex and difficult to interpret models. As a result, recent publications^49,50^ have insisted on the importance of focusing on the elements enabling CNNs to make their decisions. In this context, Wöber et al.^20^ used two approaches [Layer-Wise Relevance Propagation^51^, and Grad-CAM^52^] to provide quantitative assessments at the pixel level about how different regions of a sample image contribute to the calculated predictions. We thus show that it was possible to go further by directly generating a global activation map of all pathological individuals to crush interindividual variability and highlight the biomarkers of a population.

The generation of a global map was made possible in particular by: (1) a preprocessing step, which consisted of aligning the images, avoiding a prediction based on noise due to rotation or offset, and (2) the superposition of the maps of individual salience, minimizing the impact of individual variability due to various parameters such as age, gender, ethnicity, etc.

Another contribution of our work made possible by the use of global activation maps is the ability to quantify the areas identified by the classifier using two complementary metrics (“classes score” and “hot surface”). This contribution is important because it offers a solution to the often qualitative approach of extracting relevant features from images. In the framework of our study, it permitted, for exemple, to carry out a statistical examination of the evolution of the structures involved in retrognathia, according to the severity of the malocclusion.

The methodology we propose offers perspectives on further studies, complementary to our present work. For example, as we have already indicated, numerous studies have highlighted a relationship between facial morphology and ethnicity^6^. Therefore, it is important to use normative cephalometric data specific to the patient's ethnicity, to ensure that the measurements are interpreted correctly and that the patient receives the most appropriate orthodontic treatment. Our study was carried out retrospectively using anonymized data which do not contain information on the ethnic origin of the patients. However, we can hypothesize that the dataset we used mainly represented a French population residing in France and receiving long-term treatment for retrognathia. In addition, our methodology which consists of superimposing individual saliency maps in order to eliminate individual variability has certainly contributed to reducing the impact of individuals who are differentiated by their ethnic origin. However, it would be of great interest to participate in the debate on the impact of ethnic origin on the shape of the skull by comparing global activation maps of individuals (suffering or not from mandibular retrognathia) from different ethnic groups. In the same way, progress in the field of three-dimensional (3D) surface imaging brings new perspectives to the field of orthodontics. Techniques such as surface laser scanning and stereophotogrammetry allow the description and comparison of 3D surfaces of the face, the study of soft tissue growth, the planning of therapy, and even the evaluation of the results of orthodontic treatment or the simulation of surgical treatments^53^. In recent years, three-dimensional (3D) CNNs have been applied to medical image analysis^54^. These methods could also constitute a promising perspective of our work insofar as they have already proved to be of interest in our scientific field. Indeed Kaźmierczak et al.^55^ adopted this approach to obtain a good prediction of the direction of facial growth in patients with malocclusions, and consequently to provide a more personalised treatment and increased chances of success.

### Impact of retrognathia on the craniofacial morphology

In the field of orthodontic practice, our methodology has made it possible: (1) to identify the structures already known in literature as being impacted by retrognathia (frontal sinus, the base of the skull, the sphenoid and the cervical vertebrae); (2) to identify a structure that had not been identified by other methods (i.e., the parietal bone); (3) to quantify the dynamic evolution of the structures of the skulls involved according to the severity of retrognathia.

Many studies have sought to identify the impact of C2Rm on skull shape in order to predict mandibular growth pattern. Indeed, growth prediction plays an important role in the accurate diagnosis and treatment planning of orthodontic patients. As highlighted by Tehranchi et al.^17^, the growth pattern of the mandible, maxilla, and other craniofacial structures is critical in determining the time of onset, the duration and the prognosis of malocclusions. But in the absence of a methodology allowing a global approach, these studies were carried out using correlational methods aimed at identifying the correlations between the mandible and structures such as the cervical vertebrae^18^, the cranial base^19^ or the frontal sinus^17^. Our study is, therefore, the first to use AI as a means to globally assess the structures impacted by retrognathia by circumventing the limitations inherent in the use of landmarks. The fact that we find the results already obtained using correlational methods argues in favor of the relevance of our approach aimed at identifying common characteristics of a population by the superposition of individual saliency maps. Similarly, the identification of a new structure (parietal bone) can be explained by the fact that the cephalometric and Procrustes methods require a pre-definition of the structures to be measured, and that this specific structure had not been considered, a priori, to be relevant by experts.

In addition, in our study, the extraction of saliency maps according to the level of severity of the pathology made it possible to visualize the global dynamics of the evolution of the structures impacted by retrognathia. This behavior resulted in a significant “progressive inflammation” of the class score and an extension of the hot surface on a gravity-dependent axis including cervical vertebrae, the base of the skull and the frontal sinus. Moreover, the analyses showed that these kinetics were homogeneous for all the structures identified both for the class score and for the hot surface.

These results (taking into account the severity of retrognathia) must be considered with caution to the extent that the database did not allow us to identify the characteristics of the samples in terms of age distribution for each level of severity of retrognathia. However, a study conducted on school children aged 9 and 12 (in India) reached the conclusion that the severity of the malocclusions was higher in the 12-year-old age group than in the 9-year-old age group^56^. Despite these limitations (which have to be considered in future studies) our study confirmed an overall amplification of cranial dysmorphism (in terms of both intensity and amplitude) according to the severity of the pathology, and the involvement of frontal sinus and parietal bone from the early stages of the pathology. Further studies will be necessary to clarify the role of these two structures in mandibular retrognathia, but hypotheses can nevertheless be put forward. The frontal sinus receives the pressure of the occlusive forces through the pillars 3 (canine-canine) and constitutes a pneumatic space of adsorption. It, therefore, appears relevant that a modification of the occlusive forces as generated by the C2RM has repercussions on the sinus, an hypothesis already raised by Lieberman et al.^57^. The parietal bone is a point of convergence of the arcs of biomechanical forces of the skull. As a result, and as for the frontal sinus, it seems relevant that the alteration of the occlusive forces has an impact on its deployment and, consequently, on its morphology. Whatever the case may be, mandibular retrognathia sometimes requires, during childhood, a therapeutic intervention (for mandibular stimulation) which must necessarily take place during the peak of bone maturation. After this critical period, the only possible solution is surgery. It is, therefore, essential to be able to anticipate early (before the critical period) the need of a therapeutic intervention. In this context, the frontal sinuses and the parietal bone could constitute early predictive biomarkers of the evolution of retrognathia, and subsequent studies of the longitudinal type would be extremely relevant to test this hypothesis. It is also important to note that studying the interaction between craniofacial structures through the implementation of an integrated approach has been highlighted as a challenge by Lieberman et al.^57^. For these authors, “it is difficult to define or assess quantitative hypotheses of craniofacial integration because we know so little about the extent and nature of the many interactions likely to occur between cranial regions”. The MIE-ICNN approach we propose provides solutions to capture covariations involving different craniofacial structures and presents saliency maps as a new morphometric assessment tool that could be applied to many situations involving the extraction of relevant information in medical images.

### Parietal bone and modifications of craniofacial morphology during human evolution

According to our results, C2Rm, which is a pathology of the jaws, has an impact on the lower face (jaws and facial mass), but also on the cranial vault at the level of the parietal bone.

The frontal sinus (via the juxtaposed supraorbital torus) and, especially, the parietal bone are markers of paleoanthropological rusticity. The parietal bone has been identified as one of the key areas in the modification and gracialisation of the craniofacial architecture during evolution^58^. By demonstrating a link between malocclusion (retrognathia) and parietal bone, our study opens the way to reconsidering the masticatory theory through the hypothesis of a more extensive impact on craniofacial architecture than what is currently admitted (influence of retrognathia on changes in the shape of the skull, via the parietal bone).

Reconsidering the influence of mastication on the modification of the craniofacial phenotype during human evolution makes it possible to highlight the role of local modulating factors due to terrain and eating behaviors (such as changes in lifestyle, or in the way of preparing food, etc.). It also circumvents some of the explanatory limitations of classical models based on morphology or genetics that underestimate the role of local factors in establishing phylogenetic models.

Indeed, a progressive reduction of the mandible has taken place during human evolution, and this transition from prognathism to increasingly accentuated retrognathism has been accompanied by a series of craniofacial modifications^11^. For example, the passage from the Mesolithic to the Neolithic saw the emergence of a gracialisation of the craniofacial morphotype in the absence of any genetic modification. This passage was characterized by a transition from a hunter-gatherer to an agro-pastoralist lifestyle. The change in diet resulted in a sophistication of food preparation (grinding, cooking, soaking, leaching) and a much smoother texture than in the era of hunter-gatherers. A gracile morphotype appeared, mainly characterized by a flattening of the face and an increase in cranial capacities. The concomitance of gracile morphotype and dietary change had already led some authors to put forward the hypothesis of a masticatory influence, in particular on the projection of the face, but this hypothesis was not sufficient to account for changes in craniofacial morphology.

Our results shed new light on the debate on the development of cranial shape during hominin evolution. They could indeed provide more complete explanatory hypotheses (involving the parietal bone) on links between the increase in retrognathia and cranio-facial modifications during the evolution of hominins. However, these results are still be regarded with caution and should give rise to further investigations, given that we lack information on the average age of the two groups considered (Class I and Class II) and that the identification of the the parietal bone could be accentuated by an age-related effect.

## Conclusion

Even if the approach we propose seems promising for future investigations centered on medical images, our study is subject to significant limitations, which must be duly considered and discussed. The retrospective design may introduce biases, especially since half of the initial dataset was rejected to allow an optimal extraction of mean saliency maps. In addition, the data we used was anonymized and did not contain information on the ethnic origin of patients. However, as already mentioned, ethnic origin has a significant impact on the shape of the skull, and this information must be taken into account when carrying out a diagnosis of retrognathia and implementing the appropriate treatment. Similarly, our anonymized database did not contain information on sample characteristics in terms of age and sex, variables that may also affect retrognathia. Even if our methodology using an overlay technique to extract an average saliency map helped to reduce the impact of these different factors on variations in skull shape, these factors should be taken into account in subsequent studies in order to improve the predictive value of the results. Finally, our study was conducted on a relatively small sample, especially with regard to the effect of the severity of retrognathia, which means that its results may not be generalizable to a larger population. Further studies will be necessary to circumvent these problems and to define more precisely the benefits and limits as well as the fields of application of these innovative approaches based on deep learning and interpretable AI.

## Data Availability

Due to the medical nature of the research, data are not publicly available, in compliance with the French Data Protection Act. The framework created during the study can be made available on reasonable request by contacting the corresponding author.
